# Practical advice for successful clinical treatment with resin-bonded bridges

**DOI:** 10.1038/s41415-023-6332-5

**Published:** 2023-10-13

**Authors:** Stephanie King, Banoo Sood, Martin P. Ashley

**Affiliations:** 41415438528001 Consultant in Restorative Dentistry, Liverpool University Dental Hospital, Liverpool, UK; 399651459413598309054 Consultant and Honorary Professor in Restorative Dentistry, University Dental Hospital of Manchester, Manchester, United Kingdom

## Abstract

Resin-bonded bridges are one of the main options for replacing missing teeth for hypodontia patients. This technique offers several advantages for these patients, who are often young, have unrestored abutment teeth, and have had tooth positions optimised by orthodontic treatment. However, the replacement of missing teeth can be challenging due to tooth positions and anomalies of abutment tooth shape and size.

These patients are often young adults at the time of restoration, making the minimally invasive nature and predictable long-term success of resin-bonded bridges advantageous over other treatment methods.

This paper in the hypodontia themed issue discusses the importance of case selection and gives practical advice for the design and provision of resin-bonded bridges.

## Key points

- Understand how to select a case that is suitable for a resin-bonded bridge to improve the clinical outcomes.
- Review the step-by-step process for impression making and resin-bonded bridge cementation.
- Recognise the important steps required and the advice to give to the patient to improve the chance of long-term success with these restorations.

## Introduction

A resin-bonded bridge (RBB) uses resin cement to attach a metal-ceramic bridge to either one or two natural teeth to allow replacement of either one or more missing tooth with a minimally invasive technique. Less commonly, an all-ceramic bridge is used, based on clinician preference. RBBs can be considered conservative, benefiting from minimal or no tooth preparation and have become a routine treatment method for the restoration of small spaces.

Resin-bonding techniques using an indirect framework, initially to splint teeth and now predominantly to replace missing teeth, have been in use for over 50 years.^1^^,^^2^ During that time, academic understanding and clinical techniques have evolved substantially, along with improvements in both the cement and the materials used to fabricate the bridge and clinical decision-making to ensure improved outcomes.^3^^,^^4^

## Case selection

Success in the provision of RBBs is dependent on many factors, beginning at the stage of case selection.

**Fig. 1:**
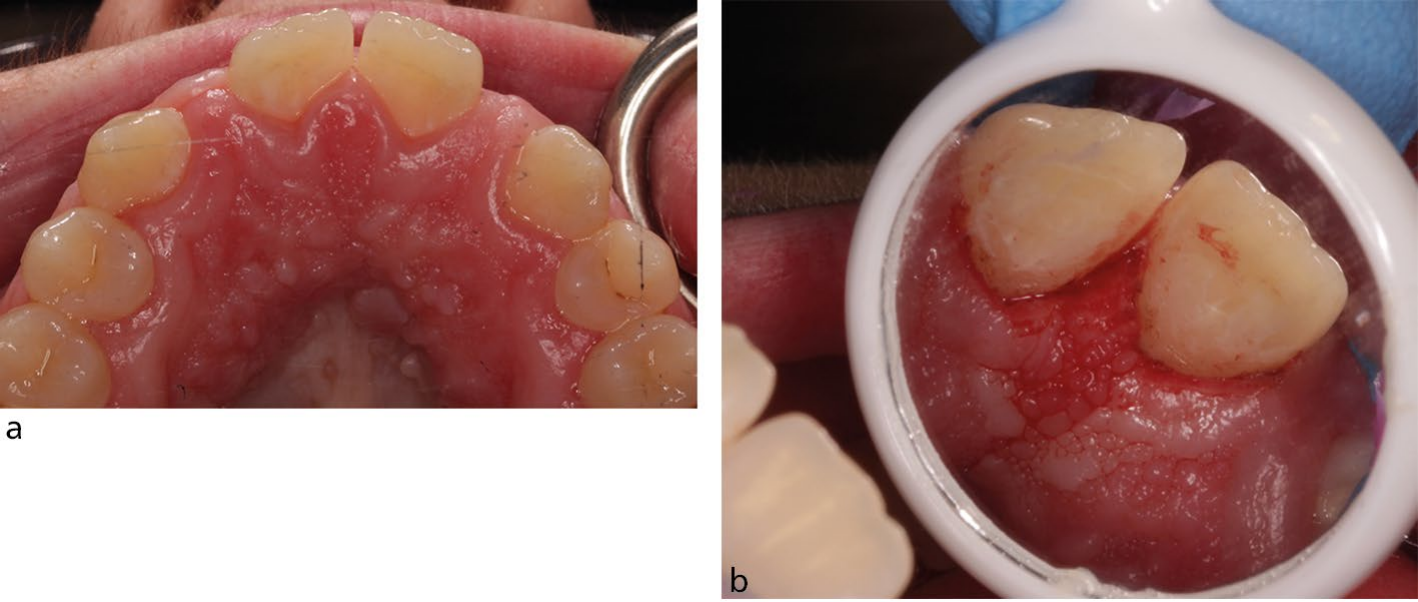
a) Hyperplastic palatal gingival tissues following prolonged orthodontic retainer use and inadequate dental hygiene. There is 'false pocketing', reducing the enamel surface area available for bonding. b) The palatal enamel surfaces have been fully exposed using an electrosurgical approach

Retention of a RBB relies on the strength of the bond between the bridge and enamel tooth surface, which is better when the abutment tooth is large and the enamel is unrestored. This can be challenging, as patients with hypodontia can also be affected by microdontia,^5^ or have hyperplastic gingival tissue from inadequate dental hygiene and long-term orthodontic retainer use. It may be necessary to use either gingival retraction cord or electrosurgery (Fig. 1) to increase the available palatal surface area of these teeth before impression making.^6^

**Fig. 2:**
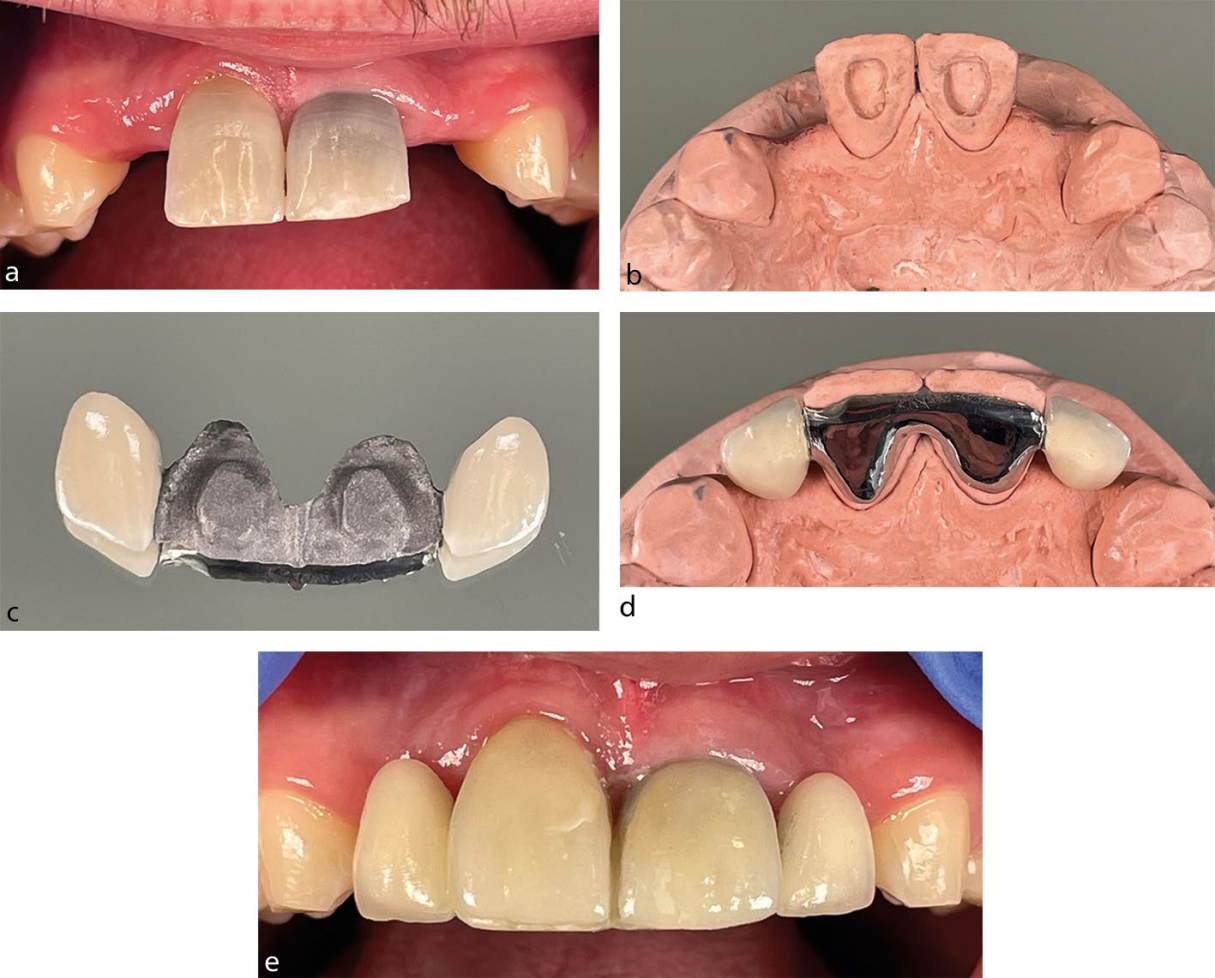
a) Hypodontia patient with endodontically treated upper central incisor teeth, requiring replacement of the missing upper lateral incisor teeth. The adjacent canine teeth have short palatal surfaces and worn tips. b) The palatal access cavities were previously restored with composite. These were reduced to reveal enamel around the periphery of each access cavity without compromising the endodontic coronal seal. c) The fitting surface of the RBB, demonstrating the access cavity preparations incorporated into the bridge structure. d) The bridge extending over as much of the enamel palatal surfaces as possible, up to but not including the translucent incisal edges. e) The RBB in place with porcelain veneers to mask the endodontic discolouration

An extensively restored abutment tooth should be avoided, as there is reduced enamel available for a successful bond. Incorporation of the existing restoration into the bridge design could be considered for a minimally restored tooth (Fig. 2).

Survival of RBBs is reduced for restored abutment teeth^4^ and the bond strength will be restricted by the weakest adherent, enamel being the strongest, with amalgam, dentine and glass ionomer much weaker.^7^ The bond-to-resin composite can be considered comparable^7^ and still have good survival if this is a new restoration.^4^ Practically, a tooth with a small restoration could still be considered a suitable abutment, provided this is changed to resin composite, ideally at the fit appointment, and all preparation margins finished on enamel.

Tooth shape and position can compromise the suitability of RBBs. An acutely angled or tilted abutment tooth may not allow sufficient connector height for the restoration, resulting in flexibility of the framework and ultimately bond failure or restoration fracture. In this circumstance, consideration would be given to either a multidisciplinary approach with the use of orthodontics or the need for a different restorative option.

Careful planning to assess the three-dimensional relationship between the space, the abutment teeth and the opposing teeth is required. On occasions, this may require articulated study casts.

A wax-up of the desired result can be used to inform the patient of the planned aesthetic outcome, potentially with options of pontic size or position.

## The clinical process

Careful completion of each of the clinical steps when providing RBBs improves the likelihood of a good outcome.

### Abutment tooth preparation

As understanding of clinical outcomes has developed, the expectation that tooth preparation is always required has changed and it can be accepted that an RBB will be successful when bonded to an unprepared enamel surface. More extensive preparation has been shown to lead to reduced survival of the bridge.^4^ However, there remain clinical situations when the enamel surface may require modification:

- To allow a path of insertion of the bridge wing over as large as surface as possible
- To allow adequate metal connector thickness between bridge wing and pontic
- To improve aesthetics by reducing black triangles or the emergence profile and correct minor misangulation of the abutment tooth
- To create a cingulum rest seat to aid bridge positioning during cementation
- To replace an unsuitable existing restoration.

### Impression making and clinical records

**Fig. 3:**
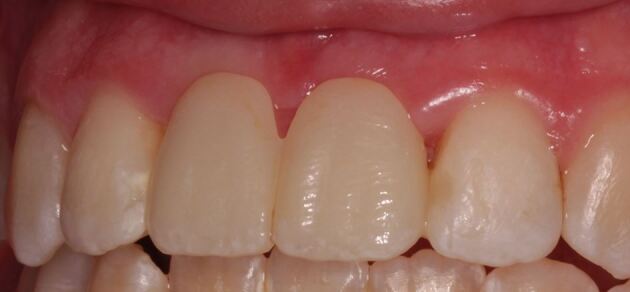
Fabrication of the RBB to replace the upper central incisor teeth, required copying of incisal opacities from the adjacent teeth
- Consider the use of gingival retraction cord if required to reveal more of the abutment tooth enamel surface
- A full arch working impression should be taken^11^ in a material that can achieve good surface detail and have sufficient dimensional stability. Commonly, this would be with a dual phase (heavy and light body or putty and wash) polyvinyl-siloxane single-stage impression. The upper impression only needs to be of a horseshoe design, which also helps patients with a gagging tendency
- Immediately before the impression material is applied to the teeth, the abutment and adjacent teeth should be thoroughly air dried to reduce the potential for air bubbles, voids and drag^11^
- A good-quality impression of the opposing arch is required. Alginate is the material of choice, as it is inexpensive, easy to use and achieves adequate surface detail. The teeth should be thoroughly dried and spread with a thin layer of alginate before the tray is inserted to ensure good occlusal detail is achieved.^12^ The alginate impression should be poured in the laboratory as soon as possible, due to the inferior dimensional stability of irreversible hydrocolloids
- An occlusal registration is recorded, using either a rigid silicone-based registration paste^13^ or a rigid dental wax, which is appropriately trimmed by the clinician to ensure that the models can be located accurately^14^
- It is important that a clear prescription is provided to the laboratory technician detailing the design of the restoration.^8^

Although there is limited evidence to favour one impression technique over another, certain principles should be adhered to, as creation of an accurate model is essential:

- Select a rigid, well-fitting impression tray, with adequate depth. If a stock tray is not suitable, a custom tray with ideal spacing and extensions around the teeth can be used
- Thoroughly clean the abutment and adjacent teeth, especially the interdental areas. If the patient does not have adequate plaque control at the planning appointment, and the abutment teeth are unclean with inflamed gingival tissues, these should be controlled by dental hygiene education and cleaning before the impression appointment
- A shade of the abutment teeth is taken ideally in natural daylight, using a tab shade guide that corresponds to the porcelain system used by the laboratory. Shade matching should take place at the beginning of the impression-making appointment to be sure that the teeth are normally hydrated. Close support from the dental technician, or communicating with them through high-quality digital photographs and detailed laboratory prescription, increases the likelihood of an acceptable outcome,^8^^,^^9^ especially as enamel anomalies are not uncommon in patients with hypodontia^10^ (Fig. 3)

### Design and construction

**Fig. 4:**
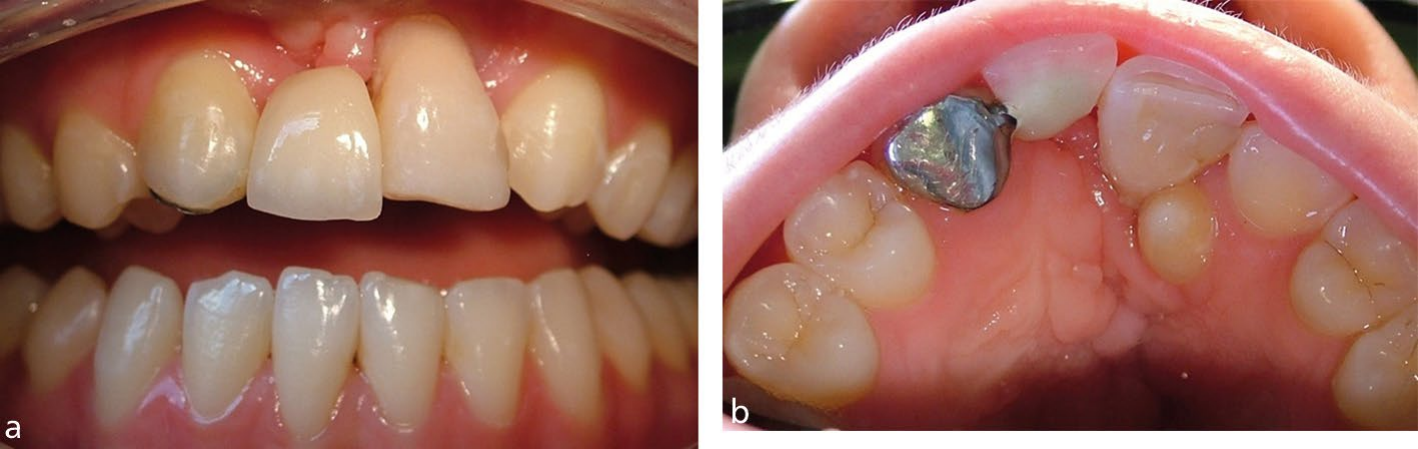
a) Cantilever RBB to replace missing upper incisor tooth. Almost three years after treatment, the patient stopped wearing the orthodontic retainer and the anterior teeth relapsed, resulting in an unattractive pontic position. b) Palatal view of the cantilever bridge after relapse of the anterior teeth. A supernumerary tooth has also erupted
- **Fixed-fixed abutment design**: Due to the success of contemporary cantilever bridges, this design is rarely indicated, other than in situations where the bridge also acts as an orthodontic retainer for teeth that are highly likely to relapse, such as after orthodontic treatment in a cleft patient (Fig. 5). Fixed-fixed bridges have the risk of debond of the minor retainer with the bridge remaining *in situ,* resulting in the potential for caries progression beneath the debonded retainer wing. However, this caries risk may not be as significant with contemporary clinical techniques as it was previously. Clinicians are less likely to select previously restored teeth as abutments and there are few situations where enamel preparation is required. A debonded bridge wing is also likely to leave some of the cement *in situ* on the enamel surface, providing some protection from the caries process

The following designs can be considered when prescribing RBBs:

- **Cantilever with a single abutment tooth**: RBBs have been shown to be most successful when a cantilever design is used and anterior bridges have been shown to be more successful than posterior bridges.^15^ However, as most hypodontia patients have had their anterior teeth repositioned before restoration, there is a risk of orthodontic relapse. While this risk is much less while the patient uses a retainer, over a period of time, most patients reduce the frequency of wearing the retainer. When a cantilever bridge is attached to a tooth that relapses, the effect on the pontic is exaggerated (Fig. 4)

**Fig. 5:**
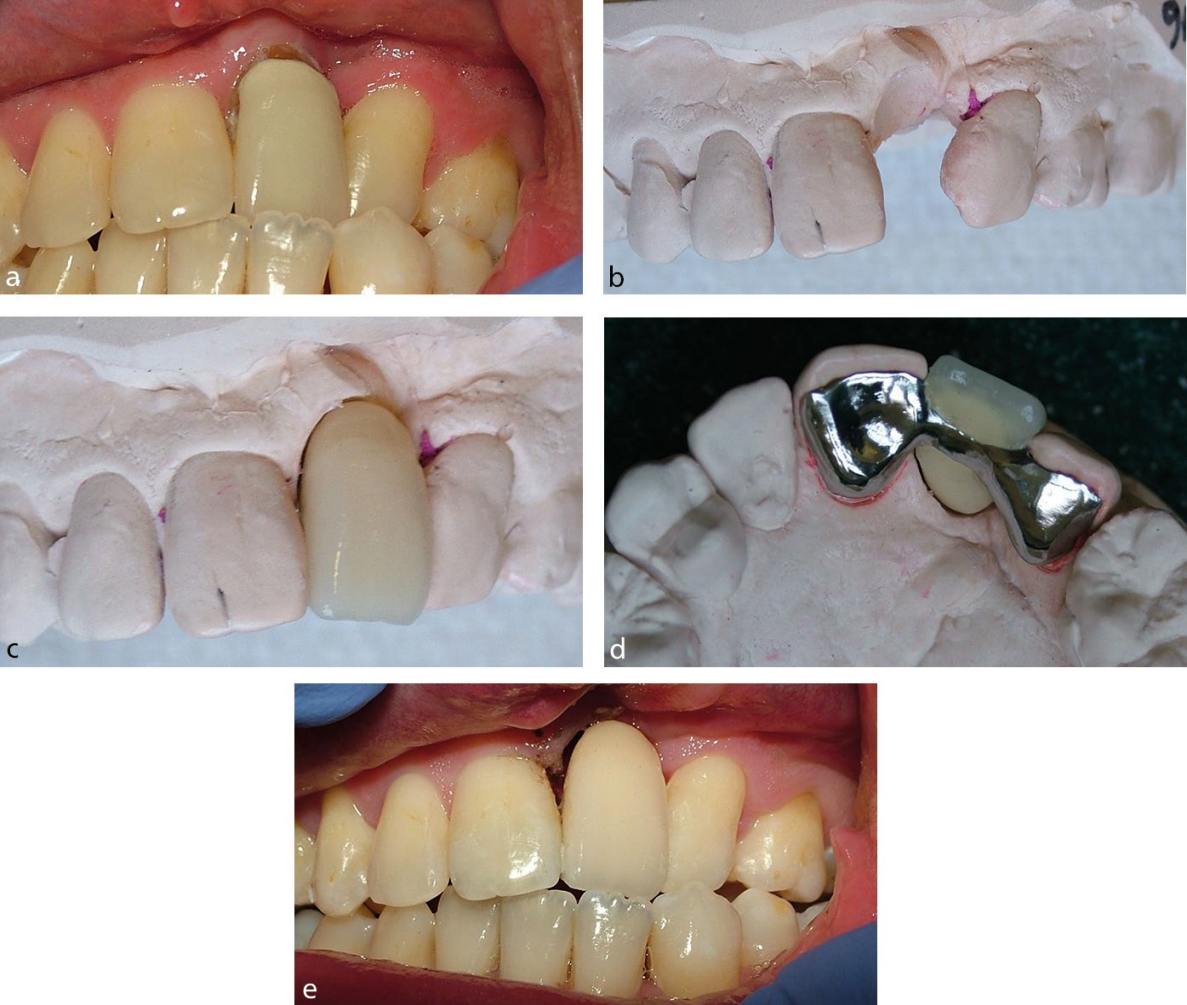
a) A cleft patient presenting with unrestorable caries of the upper left central incisor tooth and palatal relapse of the upper left dental segment. b) The model is altered to create space for an immediate replacement for the failed tooth. c) An immediate RBB is fabricated, with the pontic replicating the position of the failed tooth. d) Unusually, a fixed-fixed design is chosen to limit any further mesio-palatal relapse of the upper left canine tooth. A removable retainer is also required. e) The RBB *in situ*
- **Double cantilever with two abutment teeth**: This design is an option when replacing two missing upper lateral incisor teeth, with retainers linked and bonded to both central incisors^4^^,^^8^ (Fig. 2). This prevents unwanted relapse of a midline diastema and avoids the issue of canine rotation if these had been selected as abutment teeth for cantilever bridges.^16^

**Fig. 6:**
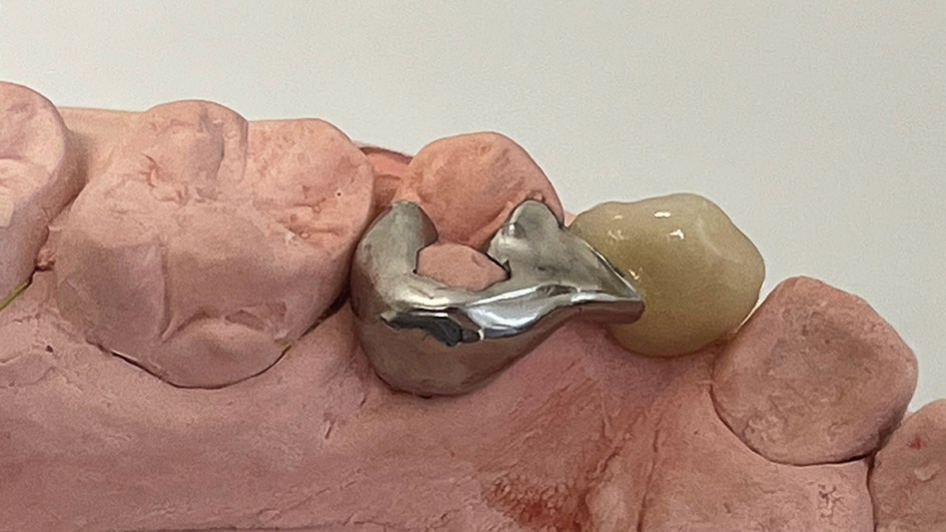
A well-designed posterior RBB with a non-perforated wing and adequately sized connector between wing and pontic. The wing wraps around onto both proximal surfaces, extends to the enamel at the cervical margin and has mesial and distal rest seats
- One or more seating hooks can be incorporated into the design of the wing to allow accurate location of the bridge and ensures a minimum thickness of resin cement^21^ (Fig. 7). The hook is removed by sectioning at the fit appointment with a diamond bur and smoothed using polishing discs with care to maintain a good finish

While it is hoped that a dental technician fabricates an RBB to an ideal design, it is the clinician's responsibility to provide a sufficiently detailed prescription to guide the dental technician's decision-making process. Practical considerations for anterior restoration design, from both technical and clinical perspectives, have been explored in another paper in this series,^8^ but to summarise the relevant clinical features:

- This should be constructed in non-precious metal alloy, with a non-perforated^17^ retainer wing and a minimum retainer thickness of 0.7 mm^18^ to ensure sufficient rigidity and minimal flexure in the framework. Although the use of all-ceramic RBBs is increasing, significant tooth preparation is advocated,^19^ and there are limitations with manufacturers only indicating zirconia for anterior RBBs
- The connector between wing and pontic should be 3 mm of metal to ensure rigidity, reducing the risk of fracture and flexion^20^
- The bridge wing should cover as much enamel surface as possible to increase retention. Cervically, this is limited by the gingival margin, but can be increased by retraction of the margin or removal of hyperplastic tissue. Laterally, this should wrap around the lingual/palatal surface as far into the interdental spaces as possible. This provides some retention form, in addition to the adhesion of the composite resin
- For anterior teeth, the wing should extend close to the incisal edge, limited by the enamel translucency. This is usually to within 2 mm of the edge but can be identified clinically by holding a dark material, such as greenstick, against the palatal surface^8^
- For posterior teeth, if there is a large lingual/palatal surface area, the wing extends close to but not over the occlusal surface. However, if there concerns about the surface area, and the cusps are in occlusion, the retention can be increased by preparing mesial and distal rest seats for the wing to engage (Fig. 6). If the cusps are not in occlusion, retention can be increased by extending the wing over at least the lingual/palatal cusps, without tooth preparation

**Fig. 7:**
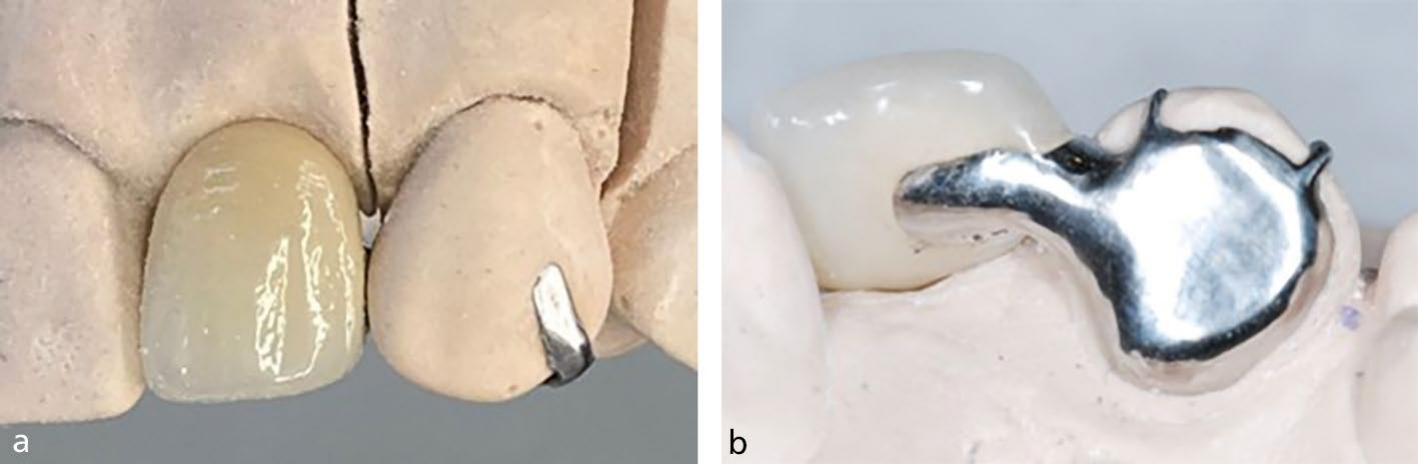
a) An RBB with a single seating hook to assist in the accurate placement during cementation. b) An RBB with a double seating hook to assist in the accurate placement during cementation
- Pontic design can vary dependent on the aesthetic demand of the case. Modified ridge lap or ovate pontics are most commonly used to achieve a good aesthetic,^21^ balanced with access to maintain adequate interdental hygiene. Pontic site soft tissue preparation may be required to achieve a higher aesthetic outcome with ovate pontics, as detailed in the literature^22^
- Occlusion must be managed precisely to ensure longevity of the restoration; this should have been examined at the assessment and may require articulated study casts so that the occlusal scheme can be fully planned. Ideally, the orthodontic outcome should have created sufficient occlusal space for both the pontic and wing. The occlusion should be designed so that there may be light contact on the pontic in the intercuspal position to act as a holding contact, but the pontic should not be involved in guidance.^10^ In situations where there is insufficient space, the bridge may be cemented 'high' so that the occlusion can re-establish over time, or subtle adjustment of the abutment and opposing teeth may be required.^23^

### Try in

The bridge is first assessed on the model to ensure it conforms to the prescribed design as communicated to the dental technician. This is also an opportunity to show and describe the features of the bridge to the patient, who is unlikely to have seen an RBB before and to describe to them how the bridge will be cemented into place.

When the bridge is held against the natural teeth, the accurate fit of the bridge can be confirmed, for both the pontic and the wing. As the bridge is unlikely to be retentive, it is necessary to hold the bridge firmly in place while observing the accurate fit against the teeth. It is also an opportunity for the clinician to rehearse how the bridge should be held in their fingers and positioned against the teeth during the cementation phase. It is not usually possible to check the occlusion at this stage or to let the patient have a full view of the bridge in place.

In addition, the shade and characteristics of the pontic tooth can be assessed. During the subsequent cementation process, the abutment teeth will dry out and subtle, short-term changes to their appearance can occur. It is worthwhile explaining this to the patient in case such slight differences are important to the patient.

**Fig. 8:**
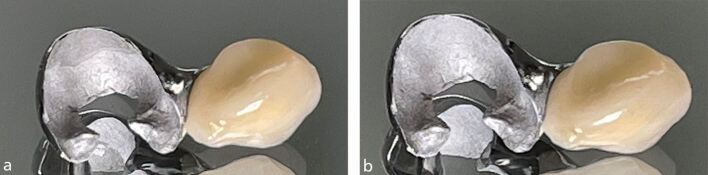
a) The bridge has been tried into the mouth, but the wing is contaminated by saliva as the abutment tooth was not adequately dry. There is a darker area on the cervical aspect of the wing, showing the contaminated surface. b) The fitting surface has been recleaned and the uniform matte appearance is restored

When trying the bridge into place in the mouth, it is important to avoid contamination of the fitting surface with moisture (saliva or blood), as this will reduce the bond strength. Therefore, the abutment teeth are cleaned and thoroughly dried first. If any contamination occurs, this will show as a change to a darker area on the uniformly matte fitting surface (Fig. 8). This is either cleaned by washing the surface with ethanol, re-sandblasting the surface (in clinic or at the dental laboratory) or by applying phosphoric acid gel for one minute and then thoroughly washing and drying.

### Fitting the bridge

#### Choice of cement

**Fig. 9:**
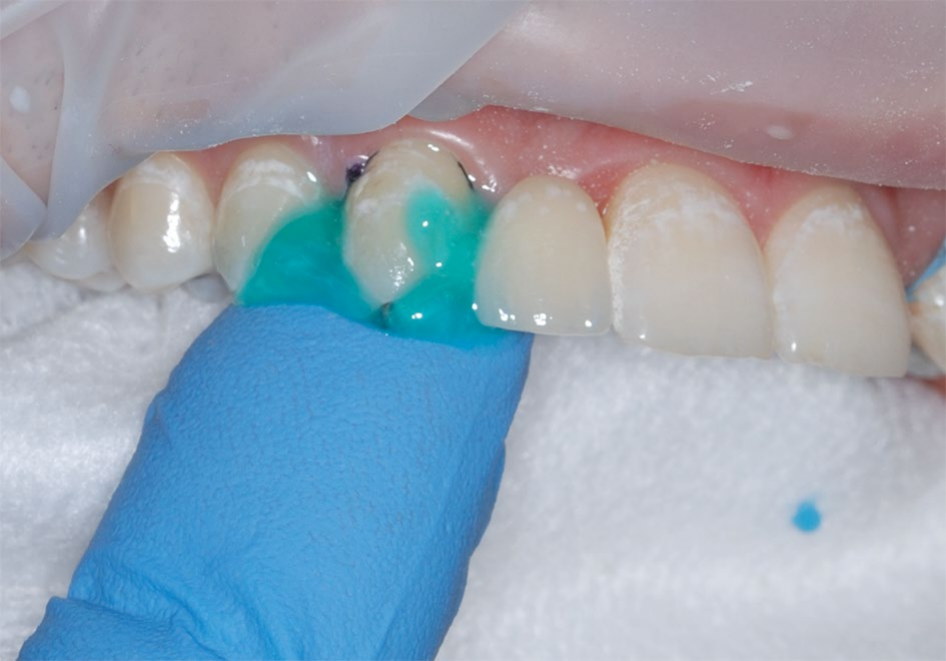
Most of the excess cement has been removed, the bridge is held firmly in place, and Oxyguard II applied, to create the anaerobic environment necessary to complete the setting process

As an RBB is designed with little, if any, retention and resistance form, it is essential that the adhesive resin cement selected is suitable to reliably bond to both the tooth structure and the non-precious metal alloy. Panavia 21 and Panavia F2.0 (Kuraray Co Ltd, Osaka, Japan) have commonly been used for this purpose for many years. These contain the MDP monomer (10-Methacryloyloxydecyl dihydrogen), capable of effective bonding to enamel, dentine and importantly, non-precious metal.^24^ The cements also have an opaque version, ideal to reduce the metal shine-through affecting incisor teeth. Panavia sets in an anaerobic environment, which is an advantage, as any excess cement in contact with the air remains unset and can be more easily removed before setting is completed by application of the Oxyguard II product (Fig. 9).

#### Communication

Regardless of which cement is preferred, the cementation process requires a number of steps to be carried out in sequence and it is essential that both the clinician and assistant understand the manufacturer's instructions and communicate with each other throughout the process. In addition, having a fully compliant patient is important and therefore, the clinician should describe each step as they occur.

#### Isolation and moisture control

As cementation requires effective moisture control, ideally rubber dam would be used to isolate the abutment and adjacent teeth. However, if the bridge wings extend to the gingival margin, it may not be possible to use rubber dam and expose sufficient enamel surface to fit the bridge. Therefore, three sources of moisture contamination of the lingual/palatal tooth surfaces are controlled:

1. Direct contact of the wet lip and tongue are prevented by use of retraction with cotton wool rolls and saliva ejector, with or without an OptraGate (Fig. 10) (Ivoclar, Liechtenstein)

**Fig. 10:**
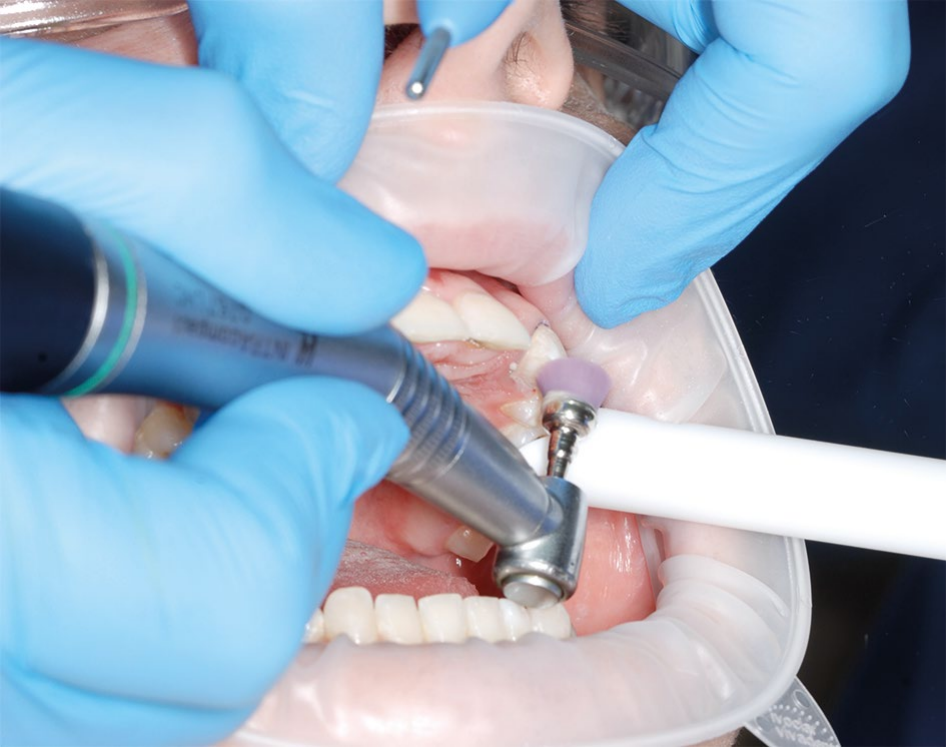
In preparation for cementing the bridge, an OptraGate has been placed and the abutment tooth is being thoroughly cleaned
2. Reduction of evaporation from the warm, moist tongue by placement of cotton gauze over the tongue. This also provides some airway protection
3. Reduction in flow of moist exhaled air by asking the patient to breathe through their nose, rather than their mouth, during the cementation process.

### Cementation process

A step-by-step cementation process when using Panavia is described in Table 1.

**Table 1:** Step-by-step techniques when cementing an RBB with Panavia

1	Allow 15 minutes for the product to reach room temperature after storage in the refrigerator
2	Ensure all components of the cement system are available in sufficient quantities to complete the process
3	Appy soft tissue retraction and moisture control methods. Clean and dry the abutment tooth surfaces (Fig. 10)
4	Place interdental separating strips between the abutment and adjacent teeth, if necessary
5	Apply phosphoric acid gel to the enamel surfaces
6	Wash the teeth thoroughly to remove the gel and cleanse the enamel surface completely
7	Change the cotton wool rolls, place a cotton gauze over the tongue and ask the patient to breathe through their nose
8	Move the overhead operating light away to prevent premature setting of the cement
9	Mix the two components of the ED Primer, dry the abutment teeth thoroughly and apply the primer to the tooth surfaces immediately, to avoid opportunities for moisture contamination. Leave for 60 seconds then dry gently. No ED Primer is applied to the bridge structure
10	Mix the two components of Panavia cement and apply a thin layer to the internal surface of the bridge
11	Seat the bridge, ensuring the abutment tooth is positioned as it was when the bridge was tried in. Maintain firm pressure for 60 seconds with gloved fingers to ensure the bridge and tooth surfaces are stable as the initial setting occurs. Some of the unset, excess cement will be removed on the glove. Most remaining cement can be carefully removed with a small brush
12	Apply Oxyguard II to the bridge margins for three minutes to ensure the cement setting continues. Wash away the Oxyguard and remove any slight excess at the margins with a probe (Fig. 9)
13	Remove the locating incisal hook, if used
14	Check the static and dynamic occlusal contacts and make any subtle adjustment to the bridge or opposing teeth as required.

### Post-cementation

**Fig. 11:**
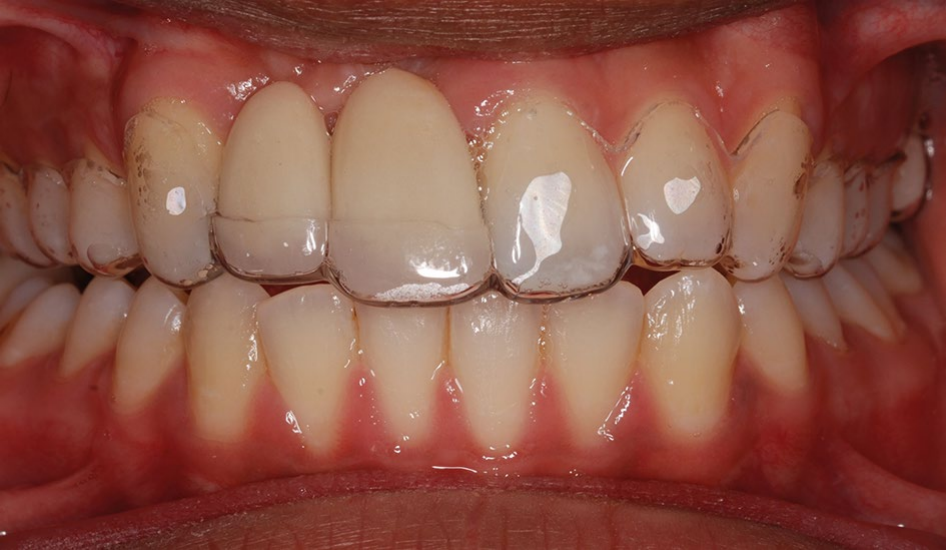
Vacuum-formed upper retainer, modified away from the cervical margin of the pontics, to reduce the force on the bridge when the retainer is placed and removed

Most young patients who require an RBB will have been wearing an orthodontic retainer that will no longer fit when the bridge is in place. Therefore, make an alginate impression for a vacuum-formed retainer that can be delivered to the patient as soon as possible, ideally within 48 hours. It is useful to have the retainer cut away from the cervical undercut of the pontic to reduce the risk of debond on insertion and removal (Fig. 11).

The patient must understand their responsibility for maintaining an adequate standard of dental hygiene each day to prevent the development of dental caries and gingival inflammation. However, it is the clinician's responsibility to demonstrate the necessary techniques and to recommend an appropriate daily hygiene regime. If interdental brushes (Te-Pe, Malmo, Sweden) or Superfloss (Oral-B, Proctor and Gamble, USA) are advised, it is probably beneficial to give a small number of these to the patient before they leave the appointment.

The patient must also understand the importance of respecting and avoiding the bridge when biting into hard or chewy food, or using their teeth for other purposes, such as biting their nails, as the patient's bite is stronger than the bridge cement.

The patient should be advised that on the rare occasion that the bridge fails and detaches from the teeth, to either revert to their original orthodontic retainer appliance, or use the new vacuum-formed retainer, with the bridge placed within it.

## Conclusion

For a young patient affected by hypodontia, there is the potential for the abutment teeth to have less available enamel surface area for bonding due to the immature gingival margin position and a translucent incisal edge on what may be a smaller than average sized tooth and therefore, there may be challenges to achieving an acceptable and reliable outcome.

However, with appropriate case selection, restoration design and good clinical technique, an RBB is a predictable, minimally invasive and well-tolerated method for replacing a missing tooth and is therefore often the restoration chosen when managing a hypodontia patient.
